# Alcohol drinking in male patients with chronic schizophrenia: prevalence and its relationship to clinical symptoms

**DOI:** 10.3389/fpsyt.2023.1164968

**Published:** 2023-07-13

**Authors:** Menghan Lv, Xuan Wang, Zhiren Wang, Xiaohong Li, Li Wang, Yunlong Tan, Xiang Yang Zhang

**Affiliations:** ^1^Beijing Huilongguan Hospital, Peking University HuiLongGuan Clinical Medical School, Beijing, China; ^2^CAS Key Laboratory of Mental Health, Institute of Psychology, Chinese Academy of Sciences, Beijing, China

**Keywords:** alcohol use disorder, positive symptoms, negative symptoms, antipsychotics, dual diagnosis

## Abstract

**Background:**

It is common practice to associate schizophrenia (SCZ) patients with substance use. The most commonly used substances in China are tobacco and alcohol. However, few studies have focused on alcohol consumption itself in patients with SCZ. Thus the purpose of this study was to detect the prevalence of alcohol use and associated clinical factors in Chinese patients with SCZ.

**Methods:**

A total of 616 male inpatients who met the Diagnostic and Statistical Manual of Mental Disorders (DSM-5) criteria for SCZ participated in this study. A detailed questionnaire, including data on alcohol consumption was used to collect demographic and clinical information on all patients. The five-factor model of the positive and negative syndrome scale (PANSS) was adopted to assess psychiatric symptoms.

**Results:**

In this study, 31.49% of SCZ inpatients had a history of alcohol use, and 82.9% of these patients abstained from alcohol use after the onset of SCZ. Compared to nondrinkers, patients who drank were more likely to smoke (*p* = 0.004), more likely to have suicide attempts (*p* = 0.002) and suicidal ideation (*p* = 0.001), more severe positive (*p* < 0.001) and depressive symptoms (*p* = 0.034), but less severe negative symptoms (*p* = 0.04).

**Conclusion:**

These findings suggest that alcohol use is common during the lifetime of SCZ patients and that alcohol use may be associated with clinical symptoms in SCZ patients.

## Background

Schizophrenia (SCZ) is a disabling psychiatric disorder with a high level of morbidity and mortality (1, 2). Patients with SCZ have a lifespan that is approximately 15 years shorter than that of general populations (3, 4). Lifestyle factors, such as poor diet, tobacco use and alcohol use, contribute not only to the high risk of medical illnesses such as cardiovascular and cerebrovascular disease (5, 6) but also to the poor outcomes of SCZ patients (7, 8), such as loss of work capacity, learning ability and social functioning. Among them, substance use is one of the factors that seriously affects the physical and psychological health of patients with schizophrenia (9–11).

Patients with psychiatric disorders are more likely to consume alcohol and become addicted compared to the general population (12). In addition, the use of alcohol and other substances may be one of the causes of SCZ (13). Several studies have shown that alcohol abuse significantly exacerbates psychiatric symptoms, increases the likelihood of relapse, decreases the benefits of antipsychotic medications, and even increases mortality in patients with schizophrenia and other psychotic disorders (14). For example, among patients with chronic SCZ, those with comorbid substance use disorders had higher positive symptom score and more severe depression than those without substance use disorders (15). Among first-episode psychosis patients, alcohol use prior to admission was associated with a higher frequency of positive symptoms (16), whereas alcohol use was associated with lower negative symptoms (17). In addition, patients with schizophrenia who drink alcohol exhibit worse cognitive function than those who do not drink (18). Alcohol consumption was associated with poorer quality of life in patients with SCZ (17, 19). Alcohol abuse disorder is also associated with elevated all-cause mortality in SCZ (20).

Prior studies have demonstrated that alcohol can promote dopamine release, and long-term alcohol use can result in down regulation of dopamine receptors, which persists even during abstinence (21, 22). There is a strong correlation between dopamine dysfunction and psychotic symptoms in SCZ (23, 24). Overall, the dopamine system may mediate the relationship between alcoholism and SCZ symptoms.

Despite the multifaceted effects of alcohol on SCZ patients, there is a lack of studies examining alcohol use in Chinese patients with SCZ. Because of the very low rate of alcohol consumption among women (15% for healthy women) compared to men (55.6% for healthy men), in the general Chinese population (25), only male patients were selected to eliminate the effect of gender differences in this study. Therefore, the aim of this study was to explore the rate of history of alcohol use in Chinese patients with SCZ, and the relationship between alcohol use history and the demographics characteristics and clinical symptoms of Chinese SCZ patients.

## Methods

All subjects selected for this study were inpatients of Beijing Huilongguan Hospital, a large psychiatric hospital in Beijing. We have determined the sample size by using the equation: *N* = (*Z*_crit_)^2^*P*(1 − *P*)/*D*^2^, where *N* is the sample size of a study group. *Z*_crit_ value is given in the table of standard normal deviations corresponding to selected significance criteria and confidence interval (CI), *Z*_crit_ value of 1.96 at 95% CI, and *D* is tolerable error, which usually takes the value of 0.1*P*. Previous data has estimated the prevalence of schizophrenia patients with a history of alcohol consumption at *P* = 36.5% (26). Then the formula yields a sample size of *N* = 668. However, some subjects had missing data on alcohol use, and finally, 616 subjects were included in this study.

The recruitment criteria adopted in this study were as follows: (1) 18 to 75 years old, Chinese male; (2) confirmation of diagnostic criteria for schizophrenia, independently assessed by one of the four psychiatrists according to the Chinese version of the Structured Clinical Interview for DSM-5 (SCID); (3) continuous type, with a disease course of at least 2 years; (4) were on stable doses of antipsychotic medication for no less than 6 months prior to their participation in the present study.

Each subject was assisted by trained researchers to complete a detailed questionnaire, including background information, social-demographic characteristics, alcohol use history, smoking status, suicidal behavior, and physical fitness.

In the inpatient setting, patients experienced a similar environment, they received three meals per day, engaged in similar activities, and were restricted from using substances other than tobacco according to a fixed schedule.

Prior to the start of this study, the Institutional Review Board of Beijing Huilongguan Hospital approved the study, and all participants signed a written informed consent form.

Using a questionnaire, frequency of alcohol consumption (days per week) and number of standard drinks consumed (10 grams of alcohol) were measured. If a person consumed no more than two drinks of alcohol per day, they were classified as moderate drinkers for the purpose of assessing psychiatric symptoms. Furthermore, binge drinking refers to consuming five or more drinks on an occasion for men. Lifetime drinkers were defined as subjects who drank alcohol at least once a week for at least 6 months (27).

A current smoker was defined as someone who smokes more than one cigarette per day in the past year (28). Suicidal ideation was defined as the occurrence of suicidal ideation or suicidal wish (29), and subjects were asked whether they had ever experienced suicidal ideation. According to the World Health Organization report, suicide attempts were defined as “a deliberate act of self-destruction, with at least some suicidal intent, but which did not result in the death of the individual” (30).

The positive and negative syndrome scale (PANSS) was adopted for the purpose of assessment psychiatric symptoms (31). The five-factor model was used to calculate the PANSS sub-scales of positive symptoms, negative symptoms, general psychopathology, depressive factor and cognitive factor (32).

PANSS was assessed by four psychiatrists specially trained in standardized use. The inter-class correlation coefficient for the PANSS was greater than 0.8 during the repeated assessment.

### Statistical analysis

Analysis of variance (ANOVA) was conducted on demographic and clinical characteristics of drinkers and nondrinkers for continuous variables and chi squared for categorical variables. Analysis of covariance (ANCOVA) were conducted to control for the effects of related variables. Additional binary regression analysis was used to assess factors related to alcohol use history. Bonferroni corrections were applied to each trial to adjust for multiple testing. Statistical significance was set at 0.05 (two-tailed), and all statistical analyses were performed by R v. 3.6.2.

## Results

Detailed types of alcohol consumption behaviors before and after illness onset are shown in Table 1. In this study, 31.49% (194/616) of male SCZ patients were lifetime drinkers. Of these drinkers, 193 patients began drinking before the onset of illness, and only one patient began drinking after the onset of illness. In addition, 82.90% (160/193) of those who drank alcohol, abstained after the onset of illness, others tended to reduce the amount of alcohol consumed.

**Table 1:** Comparison of drinking patterns in patients with schizophrenia before and after the onset of disease.

Before	After			
	No drinking	Moderate drinking	Excessive drinking	Summary
No drinking	422	1	0	423
Moderate drinking	145	30	0	175
Excessive drinking	14	1	3	18
Summary	581	32	3	616

In Table 2, we compared the demographic and clinical variables of 616 male chronic schizophrenia patients who drank alcohol and those who did not consume alcohol. There were no significant differences between the two groups on most demographic characteristics. Patients with a history of drinking were more likely to smoke, to have suicidal attempt and suicidal ideation (all *p* < 0.01) than those without a history of drinking. In addition, compared to nondrinkers, former drinkers were found to have more severe positive symptoms and depressive symptoms, while negative symptoms were less severe (all *p* < 0.05).

**Table 2:** Demographic and clinical characteristics of patients with and without history of drinking.

	Non-drinkers	Drinkers	*p*
	(*n* = 422)	(*n* = 194)	
Age	48.20 ± 13.05	47.24 ± 12.52	0.256
Education (years)	9.15 ± 2.92	9.29 ± 2.96	0.406
BMI	24.07 ± 4.15	24.66 ± 4.55	0.063
Marital status			0.718
Single	301 (71.3%)	133 (68.6%)	
Married	65 (15.4%)	31 (16.0%)	
Divorced or losing spouse	56 (13.3%)	30 (15.4%)	
Smokers/nonsmokers	157/265	96/98	0.004^**^
With/without suicide attempts	30/392	29/165	0.002^**^
With/without suicidal ideation	73/349	57/137	0.001^***^
Age at onset (years)	25.49 ± 8.04	26.37 ± 8.00	0.474
Duration of illness (years)	22.71 ± 11.86	20.88 ± 10.78	0.064
Number of hospitalizations	5.55 ± 7.86	5.21 ± 7.67	0.351
Typical/atypical antipsychotics	26/393	10/184	0.741
Anti psychotic dose (mg/day) PANSS	361.67 ± 525.75	346.42 ± 524.44	0.491
Positive symptoms	15.03 ± 4.63	16.61 ± 5.71	0.000^***^
Negative symptoms	22.00 ± 6.90	20.92 ± 6.20	0.044^*^
General psychopathology	39.22 ± 7.82	40.40 ± 8.29	0.165
Depressive factor	6.72 ± 2.32	7.16 ± 2.59	0.034^*^
Cognitive factor	9.45 ± 3.04	9.39 ± 2.97	0.338
Total score of PANSS	76.25 ± 15.52	77.93 ± 16.05	0.286

BMI, body mass index; PANSS, the positive and negative syndrome scale. ^*^Indicates that there was a significant difference between patients with and without a history of drinking. ^*^p < 0.05, ^**^p < 0.01, and ^***^p < 0.001.

After adjusting for age, disease course, and antipsychotic medications, differences in positive symptoms (*F* = 13.38, *p* = 0.000), and the depression factor (*F* = 4.44, *p* = 0.035) remained significant between the 2 groups. Only differences in suicidal ideation and positive symptoms passed Bonferroni correction. In addition, after adjusting for age, age of onset, and types of antipsychotic medication, additional binary regression analysis confirmed that lifetime alcohol use was associated with smoking status (adjusted OR = 1.84; 95% CI: 1.27–2.66; *df* = 1, *p* = 0.001), suicidal ideation (adjusted OR = 1.56; 95% CI: 1.01–2.41; df = 1, *p* = 0.057) and positive symptoms (adjusted OR = 1.06; 95% CI: 1.02–1.09; df = 1, *p* = 0.044).

Antipsychotic data were available for 616 patients, including 577 patients treated with atypical antipsychotics (clozapine 270, risperidone 101, aripiprazole 46, quetiapine 71, olanzapine 85, ziprasidone 4), 36 patients treated with typical antipsychotics (chlorpromazine 22, sulpiride 10, haloperidol 4), and 3 patients treated with unrecorded antipsychotics. No patient was on a combination of antipsychotics. The clinical characteristics of patients treated with typical and atypical anti psychotics were compared after adjusting for age and age at first hospitalization. The equivalent doses of antipsychotics were calculated by using chlorpromazine as the standard comparative drug (33). Results showed that antipsychotic doses in both groups were similar. Patients treated with atypical antipsychotics had a higher depressive factor score than those treated with typical antipsychotics (*F* = 4.82, *p* = 0.026), but there were no significant differences in any other variables between the two groups. In addition, 39.4% were on clozapine, and there were no significant differences in any variables between the clozapine and non-clozapine groups (all *p* > 0.05).

## Discussion

To the best of our knowledge, this was the first study to investigate the effects of lifetime alcohol consumption on SCZ inpatients in China. This study revealed three main findings: (1) patients with a history of alcohol use were more likely to be smokers; (2) patients with a history of alcohol use had a higher incidence of suicidal behavior; (3) the positive symptom and depressive factor scores of drinkers were significantly higher than the nondrinkers’ scores, although the negative symptom score was significantly lower than that of nondrinkers.

The current study found that 31.49% of the patients had a history of alcohol use, which is consistent with previous studies. In the general Chinese population, the current rate of male alcohol drinkers is 55.6% (25), which is significantly higher than the rate of lifetime alcohol drinkers in schizophrenia patients in this study. Zhang et al. (34) found that, 27.73% of male SCZ inpatients had a history of alcohol consumption in China whereas two other studies reported that 28%–36.5% of SCZ patients with first-episode alcohol use (26, 35). Conversely, a longitudinal study of patients with chronic SCZ reported a similar rate of alcohol consumption (34.5%) at baseline (36). A similar finding has been found in German outpatients, showing that male schizophrenia patients tend to consume less alcohol than the general population (37). In addition, we found that after the onset of illness, 82.90% of patients who drank prior to illness onset refrained from drinking. This decrease in alcohol consumption and SCZ drinking rate may be related to patients’ economic status and medical service. Harris and Edlund (38) found that mental health care users were less likely to engage in alcohol use than nonusers who may view alcohol use as a form of self-treatment. Treatment with antipsychotics may also contribute to alcohol abstinence (39). A prospective trail showed that 70% of SCZ and substance abuse patients discontinued or reduced their substance abuse after 12 weeks of clozapine treatment (40). Another reason why alcohol use may have reduced in patients with schizophrenia is their negative symptoms. In this study, those who used alcohol had lesser negative symptoms than those who did not use alcohol. This suggests that negative symptoms may have a protective effect in the case of alcohol use.

Smoking in this study was closely linked to drinking behavior. These relationships were found not only in China (34, 41), but in other countries as well (7, 19). In addition, among the general population in China, alcohol users are also more likely to smoke than nondrinkers (25, 42). This association can be attributed to shared genetic vulnerability and similar risk factors (15). Indeed, a series of studies confirmed that shared genetic factors exist between alcohol and nicotine addiction, including a study of 3,356 pairs of male twins (43, 44). Krystal et al. (45) reviewed studies and pointed out that distortions in reward processing and changes in response to substances increase the likelihood of SCZ patients with nicotine, ethanol or marijuana for self-use.

It is noteworthy that although there was a significant difference in the rates of both suicide attempts (*p* = 0.002) and suicidal ideation (*p* = 0.001) between alcohol drinkers and non-drinkers, further regression analysis showed that lifetime drinking was not significantly associated with suicidal thoughts, as the *p*-value in the regression analysis was not less than 0.05 (*p* = 0.057). However, this result suggests that lifelong drinkers were more prone to suicidal behavior (suicide attempts or thoughts) than patients with no history of alcohol consumption. Alcohol use is common among suicide attempts (46). In addition, a review of 96 studies supported the relationship between drinking history and suicide in SCZ patients (47). On the other hand, the presence of alcohol abuse disorders also increases the risk of suicide in SCZ patients (48). This relationship between alcohol consumption and suicide may be related to the function of hypothalamic pituitary adrenal (HPA) axis, which played a crucial role in suicidal behavior (49, 50). Long-term alcohol-induced changes in the intestinal microflora that regulate the HPA axis, metabolism of tryptophan precursors, and serotonin synthesis can also affect suicidal behavior (51–55).

In the current study, we found that lifetime alcohol users had fewer negative symptoms and more severe positive symptoms than those who had never consumed alcohol. Similar findings were reported in a review of SCZ patients with substance abuse (56). Dysfunction of dopaminergic neurotransmission may underlie the difference in psychopathology symptoms between alcohol-exposed and non-alcohol-exposed patients (57). Increased dopamine release from the prefrontal lobe and the mesolimbic cortex may be associated with positive symptoms, while negative symptoms are thought to be associated with a decrease in striatal and prefrontal dopaminergic release (58–60). Also, alcohol intake induces dopamine release in the striatum, and chronic alcohol consumption leads to postsynaptic downregulation, thereby modulating the function and availability of dopamine D_2_ receptors. In addition, some effects of alcohol are persistent even during abstinence (61–63).

Consistent with other studies, we found that alcohol consumption was associated with depression among SCZ patients (19, 26). Patients with atypical antipsychotics in this study had a higher level of depression than patients with typical antipsychotics, but after controlling for antipsychotic types, there was still a significant difference in depression factor scores between patients with and without a history of alcohol use. According to the self-medication hypothesis, alcohol may be used to cope with undiagnosed and potential pain, in addition to negative emotions because it provides relief from negative symptoms (38, 64). At the same time, alcohol consumption negatively affects the drinker’s marriage, economic status, and social standing, which can lead to depression (65). If this is the case, then the relationship between alcohol use and depression may be bidirectional, as supported by longitudinal studies (66, 67).

Our results are limited by several factors. First, this study adopted a cross-sectional design, which hampered the detection of alcohol use causality in SCZ. Second, the assessment of alcohol use was limited. Here, only relationships between drinking history and a variety of factors were examined. Third, some important variables that may be related to drinking, such as social-economic factors and family history of alcohol drinking, were not clearly collected. Fourth, we selected patients with chronic, stable schizophrenia as study subjects and required a constant dose of antipsychotic medication for at least 6 months prior to hospital admission. Therefore, we selected patients who were continuously hospitalized as study subjects so that the additional impact of their disease fluctuations on the study could be eliminated. However, the results of this study cannot be generalized to other SCZ populations, such as outpatients or community patients. Fifth, in this study, we only compared patients with any consumption of alcohol vs. patients who never drank alcohol. Unfortunately, we did not compare patients with alcohol use disorders to patients with moderate or low alcohol use, because we did not collect the exact amount of alcohol the patients drank. In addition, former drinkers and current drinkers were in the same group. Finally, as information on alcohol use and suicidal behavior was obtained from interviews and medical records, recall bias may exist in this study.

## Conclusion

In conclusion, our findings extend previous studies that the rate of lifetime alcohol use in SCZ patients was lower than that in the general Chinese population, and the rate of abstinence was greater than that of the general population. Patients with SCZ who had a history of alcohol use were more likely to smoke and have suicidal behavior, and they also exhibited more severe positive and depressive symptoms and less negative symptoms than patients who had never used alcohol.

## Data availability statement

The original contributions presented in the study are included in the article/supplementary material, further inquiries can be directed to the corresponding author.

## Ethics statement

The studies involving human participants were reviewed and approved by The Institutional Review Board of Beijing Huilongguan Hospital. The patients/participants provided their written informed consent to participate in this study. Written informed consent was obtained from the individual(s) for the publication of any potentially identifiable images or data included in this article.

## Author contributions

XZ: study design. ML: study conduct. XW and ZW: data analysis. LW and YT: manuscript preparation. XL: data interpretation. ML, XW, ZW, XL, LW, YT, and XZ: manuscript revision. All authors contributed to the article and approved the submitted version.

## Funding

This work was supported by CAS Pioneer Hundred Talents Program and the National Natural Science Foundation of China (81371477), Capital Health Development Research Project (2016-4-2131), Beijing Excellent Talent Cultivation Funding Project (2016000021469G174). Design of the study, collection, analysis, interpretation of data, and writing were not influenced by the funding body.

## Conflict of interest

The authors declare that the research was conducted in the absence of any commercial or financial relationships that could be construed as a potential conflict of interest.

## Publisher’s note

All claims expressed in this article are solely those of the authors and do not necessarily represent those of their affiliated organizations, or those of the publisher, the editors and the reviewers. Any product that may be evaluated in this article, or claim that may be made by its manufacturer, is not guaranteed or endorsed by the publisher.
